# Liquid-Driven Microinjection System for Precise Fundus Injection

**DOI:** 10.3390/s24072140

**Published:** 2024-03-27

**Authors:** Shiyu Xu, Bo Hu, Rongxin Liu, Xin Zhao, Mingzhu Sun

**Affiliations:** 1 National Key Laboratory of Intelligent Tracking and Forecasting for Infectious Diseases, Engineering Research Center of Trusted Behavior Intelligence, Ministry of Education, Tianjin Key Laboratory of Intelligent Robotics, Institute of Robotics and Automatic Information System, Nankai University, Tianjin 300350, China; shiyuxu@mail.nankai.edu.cn (S.X.); hubo@mail.nankai.edu.cn (B.H.); 2120230484@mail.nankai.edu.cn (R.L.); zhaoxin@nankai.edu.cn (X.Z.); 2 Institute of Intelligence Technology and Robotic Systems, Shenzhen Research Institute of Nankai University, Shenzhen 518083, China

**Keywords:** fundus injection, microinjection system, PID-SMC, microfluidics

## Abstract

Microinjection is usually applied to the treatment of some retinal disorders, such as retinal vein cannulation and displaced submacular hemorrhage. Currently, the microinjection procedure is usually performed by using the viscous fluid control of a standard vitrectomy system, which applies a fixed air pressure through foot pedal activation. The injection process with the fixed pressure is uncontrollable and lacks feedback, the high flow rate of the injected drug may cause damage to the fundus tissue. In this paper, a liquid-driven microinjection system with a flow sensor is designed and developed specifically for fundus injection. In addition, a PID sliding mode control (SMC) method is proposed to achieve precise injection in the injection system. The experimental results of fundus simulation injection demonstrate that the microinjection system meets the requirements of fundus injection and reduces the impact of the injection process on the fundus tissue.

## 1. Introduction

With the aging of the population and changes in people’s lifestyles, the global incidence of various fundus diseases is on the rise. Some of the common conditions include central retinal artery occlusion (CRAO) [1,2], central retinal vein occlusion (CRVO) [3], age-related macular degeneration (AMD) [4], and other related diseases. For CRAO/CRVO or submacular hemorrhage caused by AMD, the intra-vascular injection of tissue plasminogen activator solution (tPA) or subretinal injection of air and tPA has emerged as a new therapeutic approach [5,6]. Subretinal drug delivery involves directly injecting the drug into the subretinal space, allowing the drug to directly act on the retina and retinal pigment epithelium (RPE) [7]. For intra-vascular injection in retinal artery cannulation and retinal vein cannulation, the surgeons inject thrombolytic drugs into the occluded retinal arteries or veins to lyse the thrombus, thereby restoring blood circulation to the retinal vessels [8,9].

In clinical settings, surgeons need to precisely puncture the blood vessels or retina before smoothly injecting thrombolytic drugs. It is difficult for surgeons to meet the accuracy requirements of the procedure due to the tremor of the human hand. Surgical robots offer a viable solution for fundus disease treatment [10]. An actively stabilized, handheld 3-degrees-of-freedom (DOF) eye surgery micromanipulator has been developed at Carnegie Mellon University. It eliminated unconscious hand movements and enabled precise insertion of the injection needle into the treatment site [11]. Researchers from Vanderbilt University developed a miniature distance sensor based on optical coherence tomography (OCT) technology. The sensor can be embedded in surgical forceps. Surgeons can judge the distance of the forceps end relative to the tissue from the OCT image in front of the forceps [12]. Researchers at Johns Hopkins University affixed the Fiber Bragg grating (FBG) to the shaft of the surgical instrument and acquired the transverse and axial forces of the membrane peeling procedure [13]. These studies solve the problem of how to accurately deliver an injection needle into a blood vessel or under the retina and keep it in place. However, fundus microinjection of drugs, which is an extremely precise operation, still presents a challenge due to the small scale of the operation and the high precision of injection flow control. 

In current clinical operations or experiments, fundus microinjection is usually performed by using the vitrectomy system [14] or commercial variable-speed syringe pump [15]. The doctors or experimenters set the injection pressure by experience and use the foot pedal to start and stop the injection. This process is uncontrollable, and the impact of the injection on the fundus tissue may easily cause damage to the retina and RPE [16,17], which necessitates stricter requirements for the safe control of the flow rate.

In microinjection, syringe pumps [18], peristaltic pumps [19], and pressure-driven pumps [20] are commonly used active pumping systems in which syringe pumps are the most effective option for precise flow control. However, the syringe pumps usually use stepper motors, which may lead to mechanical vibration [21] due to the factors of ball screws or friction resulting from inadequate maintenance. In particular, the flow rate fluctuations caused by mechanical vibration may result in droplet generation when controlling small flow rates [22]. In addition, in surgical robots for fundus microinjection, the injection mechanism should be fixed to the surgical arm of the surgical robot platform [23,24,25], necessitating a small size and a lightweight mechanism. The common mechanical knob-type syringe pumps are large with a long stroke [26], making them unsuitable for fundus microinjection.

In this paper, we design and develop a liquid-driven microinjection system for the fundus microinjection surgical robot. Unlike the traditional rigid mechanism connected to the piston, the driver pushes the silicone oil and the silicone oil drives the piston in the proposed system to inject the drug. This liquid-driven method provides the liquid buffer for each forward step of the motor, which effectively reduces flow shock due to motor steps. In addition, the proposed system is smaller in size compared to the stepper motor syringe pump, which makes it easier to fix on the surgical robot platform. The drug syringe of this system is the same as that of the standard vitrectomy system, so it can be loaded onto the robotic arm directly. Moreover, we propose a flow rate control method based on PID sliding mode control (SMC) for precise injection, particularly for the precise regulation of injecting drugs into the fundus. The experimental results of fundus simulation injection demonstrate the high efficiency and stability of the proposed injection system.

The contributions of this paper are as follows:

(1) A liquid-driven microinjection system is designed, which has a flow sensor as the feedback for closed-loop control of the flow rate. The liquid-driven injection method proposed in this paper has better controllability compared to gas-driven injection. Meanwhile, the weight applied to the robotic arm can be reduced with this method.
(2) A PID-SMC method is proposed to achieve precise control of drug microinjection. This approach reduces overshooting and improves the precision and robustness of the system compared to traditional control methods.
(3) A fundus experimental environment is designed to simulate fundus pressure in microinjection. In this environment, tracking experiments with different flow rates are carried out to verify the feasibility of the designed system and the proposed control method.

## 2. Materials and Methods

### 2.1. Design and Modeling of Liquid-Driven Microinjection System

#### 2.1.1. Design of Liquid-Driven Injection System

**Figure 1:**
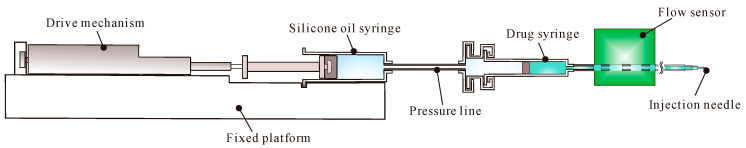
Design of Liquid-Driven Injection System.

To achieve precise control of drug injection, we developed a liquid-driven injection system. Figure 1 shows the overall schematic diagram of the liquid-driven injection system, which includes the drive mechanism, silicone oil syringe, drug syringe, flow sensor, and pressure pipeline. The drive mechanism is connected to the silicone oil syringe via a mechanical mechanism. The silicone oil syringe is then connected to the drug syringe via a pressure pipeline. The drug syringe is connected to the flow sensor through the drug delivery pipeline. Finally, it is connected to the injection needle.

In this system, the front of the drug syringe is filled with drug solution, while the back end of the drug syringe, pressure pipeline, and silicone oil syringe are filled with silicone oil. When the injection is performed, the drive mechanism is controlled to push the piston of the silicone oil syringe forward, the silicone oil then has a certain pressure to push the drug piston, causing the drug to be injected.

The fluid-driven approach injects the drug indirectly. The drug fluid reaches the injection needle through a line. Thus, the injection needle, weighing a few grams, is fixed to the robotic arm of the surgical robot. This replaces the motor and syringe mounted on the robotic arm [27], greatly reducing the loading of the robotic arm.

The proposed liquid-driven injection system has the following advantages:

(1) The liquid-driven device indirectly propels the drug piston, thereby reducing flow fluctuations caused by the motor pulse.
(2) The injection system extends the injection needle to the robotic arm, enabling integration of the injection mechanism into the surgical robot’s operating arm.
(3) Compared to the standard vitrectomy system for fundus injection, this system has a feedback flow control which makes the flow output smoother.

#### 2.1.2. Modeling of Liquid-Driven Injection System

**Figure 2:**
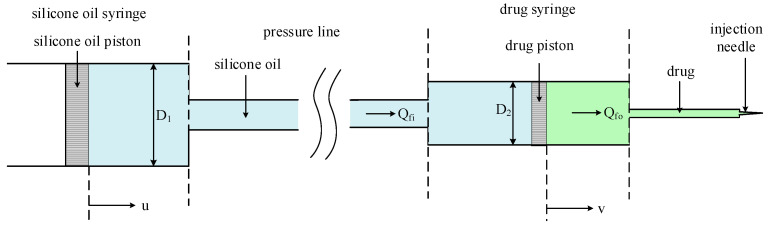
Modeling of Liquid-Driven Injection System.

The modeling of the liquid-driven injection system is shown in Figure 2. Due to the transient response of the piston’s rubber, the piston velocity is not strictly equal to the velocity of the fluid. As for the silicone oil syringe, the relationship between the velocity of the silicone oil piston u and the velocity of the silicone oil $v_{fi}$ can be considered a first-order system [28]:

(1) $$\tau^{\prime }{\dot{v}}_{fi}+v_{fi}=u+{d_{Q}}^{\prime }$$

where $\tau^{\prime }$ is the time constant of the injection model and ${d_{Q}}^{\prime }$ is the unmodeled perturbation.

Flow rate is the volume of liquid through the pipeline in a certain period of time. Without taking into account the pipeline wall of the residual and leakage losses, the flow rate in different diameters of the pipeline is constant, and its relationship with the velocity of the fluid is proportional [29]. The proportional constant is the cross-sectional area of the pipeline through which the liquid passes. The flow rate of the silicone oil $Q_{fi}$ can be expressed as:

(2) $$Q_{fi}=S_{1}v_{fi\;},S_{1}=\frac{\pi {D_{1}}^{2}}{4}$$

where $S_{1}$ is the cross-sectional area of the silicone oil syringe, $D_{1}$ is the diameter of the silicone oil syringe. 

Substituting Equation (2) into Equation (1), we have:

(3) $$\frac{4}{\pi {D_{1}}^{2}}\left(\tau^{\prime }{\dot{Q}}_{fi}+Q_{fi}\right)=u+{d_{Q}}^{\prime }$$

Similarly, the piston velocity of the drug syringe v and the flow rate of the drug $Q_{fo}$ should also satisfy the relationship of a first-order system:

(4) $$\frac{1}{S_{2}}\left(\tau {\dot{Q}}_{fo}+Q_{fo}\right)=v+d_{Q},\;S_{2}=\frac{\pi {D_{2}}^{2}}{4}$$

where τ is the time constant of the injection model, $d_{Q}$ is the unmodeled perturbation, $S_{2}$ is the cross-sectional area of the drug syringe, and $D_{2}$ is the diameter of the drug syringe.

Since the volume of the pipeline is relatively small and the viscous fluid is essentially incompressible, the actuation of the viscous fluid has less effect on the motion of the syringe piston. In addition, the brushless servo motor has a high-precision closed-loop controller integrated inside. It can precisely output the speed of pushing. Thus, the uncertainty and nonlinearity of the injection system can be dealt with through feedback controllers. Combining the above considerations, in order to simplify the model to facilitate analysis and control, the syringe piston velocity v and the flow rate of silicone oil $Q_{fi}$ have the following relationship [30]:

(5) $$Q_{fi}=S_{2}v+d_{q}$$

where $d_{q}$ is the centralized uncertainty caused by the simplification of the dynamic model and other unmodeled disturbances.

Combining Equation (5) with Equation (4) and removing the intermediate variable v, the relationship between $Q_{fi}$ and $Q_{fo}$ can be obtained. Then, substituting $Q_{fo}$ into Equation (3), the flow rate of the drug $Q_{fo}$ can be expressed as:

(6) $${\ddot{Q}}_{fo}=Au+B{\dot{Q}}_{fo}+CQ_{fo}+F$$

where *A*, *B*, and *C* are represented as:

(7) $$\left\{\begin{array}{c} A=\frac{\pi {D_{1}}^{2}}{4\tau \tau^{\prime }}\\ B=-\frac{\tau +\tau^{\prime }}{\tau \tau^{\prime }}\\ C=-\frac{1}{\tau \tau^{\prime }} \end{array}\right.$$

F is a lumped disturbance, which has an upper bound that satisfies:

(8) $$\left|\left|F\right|\right|\leq \alpha +\beta \leq \xi$$

where α is an upper bound on the nonlinear perturbation, β is an upper bound on the unmodeled part of the perturbation, and ξ is an upper bound on the overall perturbation.

### 2.2. Flow Rate Control Based on PID-SMC

#### 2.2.1. PID SMC Method

In this study, SMC is utilized to control the flow rate within the required error according to the characteristics of the system. The error is defined based on the flow rate of the drug:

(9) $$e=Q_{fo}-Q_{d}$$

where $Q_{d}$ is the desired flow rate of the drug.

To design the control law applicable to the system with zero tracking error, the sliding mode surface should satisfy the following conditions:

(10) $$s=k_{1}e+k_{2}\int edt+\dot{e}$$

where $k_{p}$, $k_{i}$, and $k_{d}$ are positive gain coefficients.

To make the system to be asymptotically stabilized, the exponential convergence rate is chosen for the sliding mode surface:

(11) $$\dot{s}=-ws-Wsat\left(s\right)$$

where w is the positive control gain, which indicates the convergence speed of the tracking sliding mode surface. W is the positive high-frequency switching gain, and sat(s) is a switching function, which is defined as follows:

(12) $$sat\left(s\right)=\left\{\begin{array}{c} 1,\\ \frac{s}{\varphi },\\ -1, \end{array}\right.\begin{array}{cc} & s>\varphi \\ & \left|s\right|\leq \varphi \\ & s<-\varphi \end{array}$$

According to Equations (9)–(11), the desired control law is constructed as:

(13) $$u=\frac{1}{A}\left(B{\dot{Q}}_{fo}+CQ_{fo}+{\ddot{Q}}_{d}+F+-k_{1}\dot{e}-k_{2}e-ws-Wsat\left(s\right)\right)$$

where the high-frequency switching gain W should be greater than the upper bound of the overall perturbation and should satisfy the following conditions:

(14) W≤ξ

To prove the validity of the proposed control law, the positive definite Lyapunov function is chosen:

(15) $$V=\frac{1}{2}s^{2}$$

Then we have:

(16) $$\begin{array}{c} \dot{V}=s\dot{s}\\ =s\left(k_{1}\dot{e}+k_{2}e+\ddot{e}\right)\\ =s\left(k_{1}\dot{e}+k_{2}e+Au-B{\dot{Q}}_{fo}-CQ_{fo}-{\ddot{Q}}_{d}-F\right). \end{array}$$

According to Equations (13) and (16), we have:

(17) $$\dot{V}=-s\left(ws+Wsat\left(s\right)\right)=-ws^{2}-Wsat\left(s\right)s$$

Since both w and W are positive, and it is easy to calculate that sat(s)s≥0, Equation (17) is a negative definite function and, therefore, satisfies the Lyapunov stability criterion.

#### 2.2.2. Simulation

**Figure 3:**
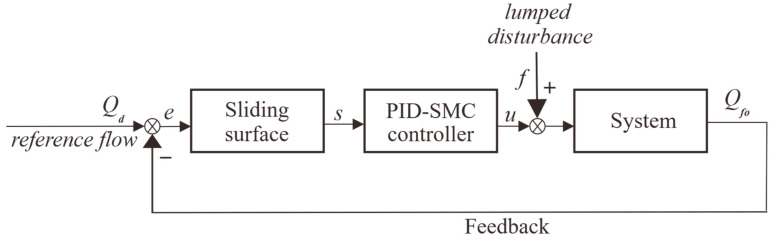
System control framework.

Before applying the proposed control algorithm to the injection system, simulations are carried out to verify its performance. The system model and controller were built by Matlab/Simulink, as shown in Figure 3.

The microinjection system should be applicable to various injection environments. Different injection durations and drug volumes are required for different fundus diseases. The volume of the liquid to be injected depends on the aim and content of the subretinal injection, such as tPA, adenovirus vector, cell suspension, and balanced salt solution (BSS). The duration of the injection depends on the fundus site where the injection is performed. This is because tissues in different places have different levels of tolerance for different surgical procedures. Microneedles and other equipment may cause tissue damage when the injected liquid remains in the vein or the interstitial space of the retina and RPE for an extended period.

In the data related to fundus injection procedures, it was found that 0.4 mL of tPA solution was injected for 3 min in the treatment of CRVO [5]. In another study, 0.05 mL of the drug was injected for 3 min to treat CRVO [31]. Additionally, 0.2 mL of air and 0.4 mL of tPA solution were used in the treatment of submacular hemorrhage [6]. Thus, the typical range of injection flow rates required in fundus surgery is approximately 10 µL/min to 200 µL/min, with the volume injected ranging from 50 µL to 600 µL.

According to the previously analyzed injection volume and injection duration requirements, the proposed control algorithm is validated by selecting a flow rate of 100 μL/min.

In the simulation, the model parameters are set to A=46.79,B=2.6,C=6, and the control gain is selected as $k_{p}=7,\;k_{i}=0.05,\;k_{1}=8.5,\;k_{2}=1$ by using system identification. The proposed controller is compared with the PID controller. The PID control gain is set to ${k_{p}}^{\prime }=0.35,\;{k_{i}}^{\prime }=0.4,\;{k_{d}}^{\prime }=0.1$.

In order to simulate the interference of the experimental environment and the measurement interference of the fluid sensor, a noise signal with the normal distribution (sampling time of 0.04 and average amplitude of 5) is added to the output of the controller and the measurement of the flow rate, respectively.

**Figure 4:**
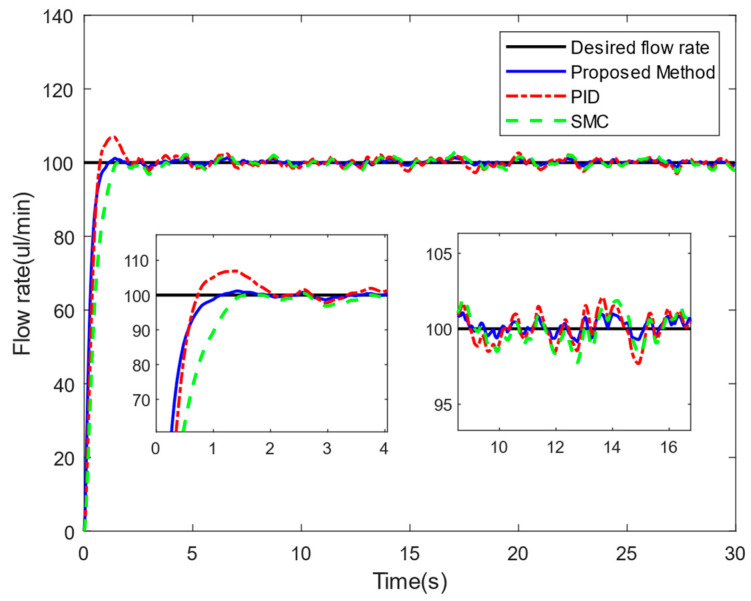
Simulation results.

The simulation results are shown in Figure 4. Compared to PID, the proposed PID-SMC converges faster to different desired positions with higher accuracy. And there is basically no overshoot for PID-SMC.

### 2.3. Injection Pressure Analysis Modeling

**Figure 5:**
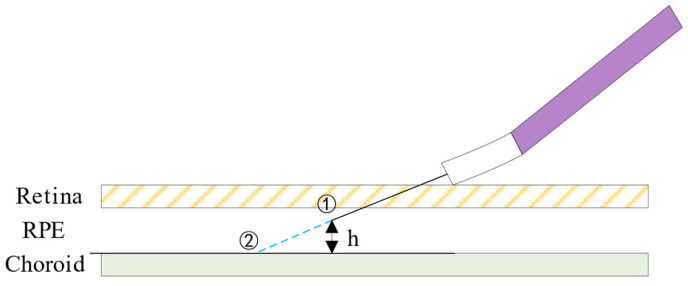
Modeling of the injection impact process.

The large overshoot flow rate in the injection will cause a lot of pressure on the fundus tissue. To quantify the tissue damage caused by the high-flow impact, we analyzed the injection pressure on the needle site between the retina and the choroid by using the Bernoulli equation [32], as shown in Figure 5.

In this study, the Bernoulli equation is applied under the assumptions of a steady state, incompressibility, frictionless flow, and a single streamline. It is also assumed that there is no external transfer of energy or heat. It can be modeled as follows:

(18) $$\begin{array}{c} p_{1}+\frac{{\rho V_{1}}^{2}}{2}+\rho gZ_{1}=p_{2}+\frac{{\rho V_{2}}^{2}}{2}+\rho gZ_{2}\\ Q_{fo}=V_{1}A_{1} \end{array}$$

The subscript 1 in Equation (18) refers to the top of the injection needle, corresponding to point ① in Figure 5. The subscript 2 refers to the impacted surface of the choroid, corresponding to point ② in Figure 5.

$P_{1}$ represents the pressure at the injection position out to the RPE, which is equivalent to the intraocular pressure of the eyeball in the injection state, and it is set to $P_{1}$ = 20 mmHg. $V_{1}$ can be calculated by using the injection flow and the area of the inner diameter of the injection needle. $Z_{1}$ is the vertical distance between the choroid and the end of the injection needle, which can be taken as $Z_{1}$ = 25 μm. $P_{2}$ represents the pressure at the point where the drug is injected into the choroid. This is the maximum impact pressure we need to calculate to get. $V_{2}$ represents the velocity at the surface of the choroid. It is set to $V_{2}$ = 0 under the condition of no slippage. $Z_{2}$ is the reference plane, so $Z_{2}$ = 0. $A_{1}$ is the cross-sectional area of the injection needle.

## 3. Experimental Results

### 3.1. System Setup

In Section 2.2.2, we determined that the maximum injection volume we need is 600 μL. Therefore, the volume of the drug syringe was selected as 1 mL. The number of coding bits of the selected high-precision driver motor is 14 bits. Then, a 2.5 mL silicone oil syringe was chosen based on the drive distance for each step. It prevents frequent loading and unloading of silicone oil while ensuring accuracy, and it ensures that we can inject 600 μL and more drugs at a time.

**Figure 6:**
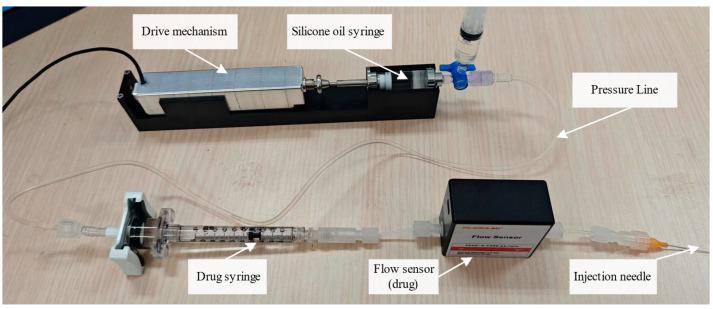
Microinjection system setup.

The primary components of the system consist of a micro-linear servo actuator (BLACF30-C112, Inspire-Robots, Beijing, China), the 2.5 mL silicone oil syringe, two flow sensors (SLI-1000, SENSIRION, Stäfa, Switzerland), a pressure pipeline (polyvinyl chloride (PVC) pipe and polytetrafluoroethylene (PTFE) pipe), and the fixed base for the injection device, as shown in Figure 6.

The pressure pipeline between the silicone oil syringe and the drug syringe is the PVC pipe. This kind of pipe effectively slows down or absorbs the amplitude of the motor pulse, which is very important for slowing down and controlling the drug flow rate. The flow sensor and the injection needle are connected by the PTFE pipe, which is more resistant to disturbances during the injection process, and can resist the disturbances caused by the sudden twisting of the tube on the flow rate during the injection process.

The two key components required to realize this hardware system are described as follows:

(a) Micro-Linear Servo-Actuator

**Figure 7:**
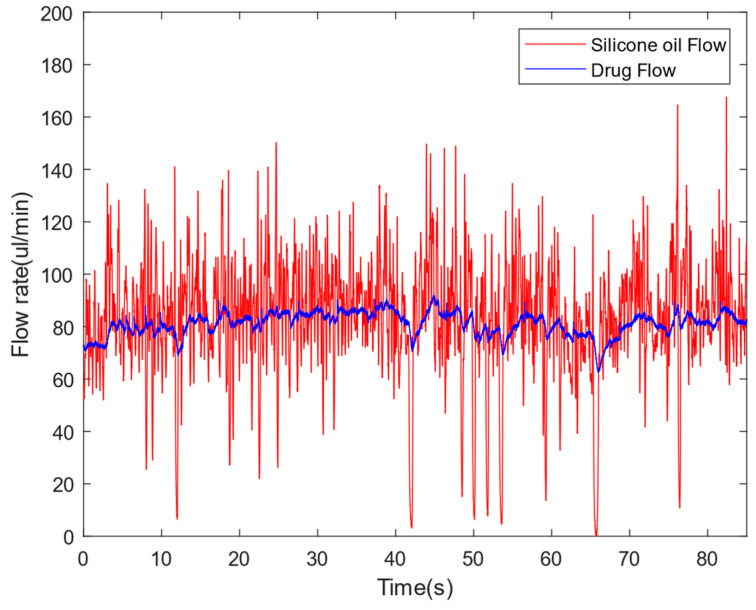
Comparison of mitigated vibration pulse flow with open loop.

The micro-linear servo-actuator is a small integrated linear servo system that integrates a core-less motor, a precision planetary reducer, a sensor, a precision screw mechanism, and a closed-loop control system. It is used to push the piston of the silicone oil syringe, with a push range of 30 mm and a maximum push–pull force of 120 N. The actuator guarantees the surgical injection volume and provides sufficient push force. Due to the buffering of the piston, the shaking of the drug flow rate can be effectively reduced. As shown in Figure 7, when a constant speed is provided to the electric cylinder in the open-loop state, the flow amplitude of the drug is significantly smaller than that of silicone oil.

In addition, a pressure sensor is configured on the rear end of the actuator, which can be used to measure the pressure applied to the actuator. This provides the possibility for more accurate control in the future.

(b) Flow Sensor

The flow sensor measures the volume of fluid flowing through a given pipe per unit of time. In this study, we used the flow sensors to accurately measure the flow rate during fundus injection. The flow sensor for silicone oil was used to measure the flow rate of silicone oil viscous fluid, while the flow sensor for the drug solution was used to measure the flow rate of the drug. The two flow sensors were calibrated by using the weighing method for the silicone oil and drug solution, respectively.

**Figure 8:**
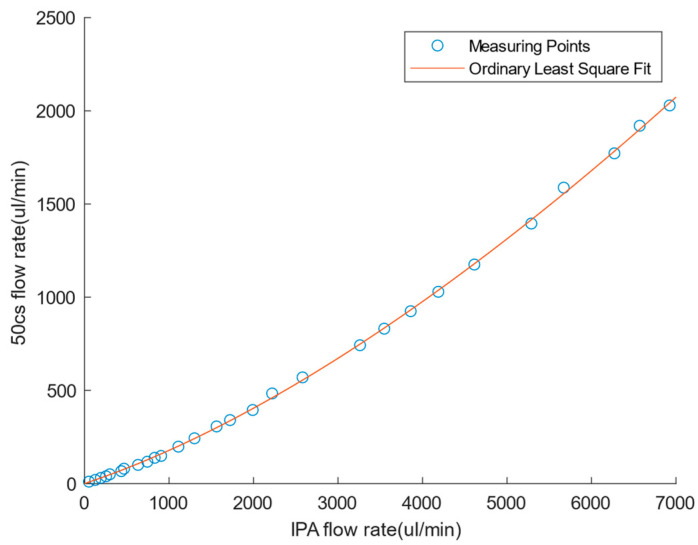
Flow Sensor Calibration.

Figure 8 shows the fitted quartic function curve for calibrating the 50c silicone oil flow rate based on the IPA solvent. The method used for fitting was the least square method. The flow rate of silicone oil was calibrated to the range of 0–2000 μL/min. This was sufficient for subsequent flow comparison experiments. Since there was little difference between the density of diluted indocyanine green solution and water in the experiment, the flow sensor standard aqueous solution mode was used directly for drug flow measurement.

The injection system should be pre-operated before the experiment. The operation steps are listed as follows:

(1) Open all three ends of the medical tee and use a syringe with 50 cs viscosity silicone oil to fill the pipeline. Remove the silicone oil syringe from the upper end of the medical tee and close the top of the tee.
(2) Use another syringe to fill the drug syringe with the desired volume of indocyanine green solution. Then, connect the drug syringe to the flow sensor through the luer.

After the preoperation, the entire pipeline is filled with 50 cs viscosity silicone oil. The drug syringe is connected to the flow sensor through the pipeline to the microneedle. The sampling time of the flow sensor is 6 ms, providing high-precision and fast flow feedback to the controller.

### 3.2. Fundus Simulation Injection Experiment

In order to verify the effectiveness and superiority of the designed injection system and control method, we performed the injection experiment in the system shown in Figure 6 and compared the proposed method with the traditional methods. The injections were completed in water at a certain depth to simulate the real-pressure environment of intraocular surgery as much as possible. Specifically, a measuring cylinder was filled with water to a depth of 272 mm and 36 °C to simulate a pressure at the fundus of 20 mmHg [33].

**Figure 9:**
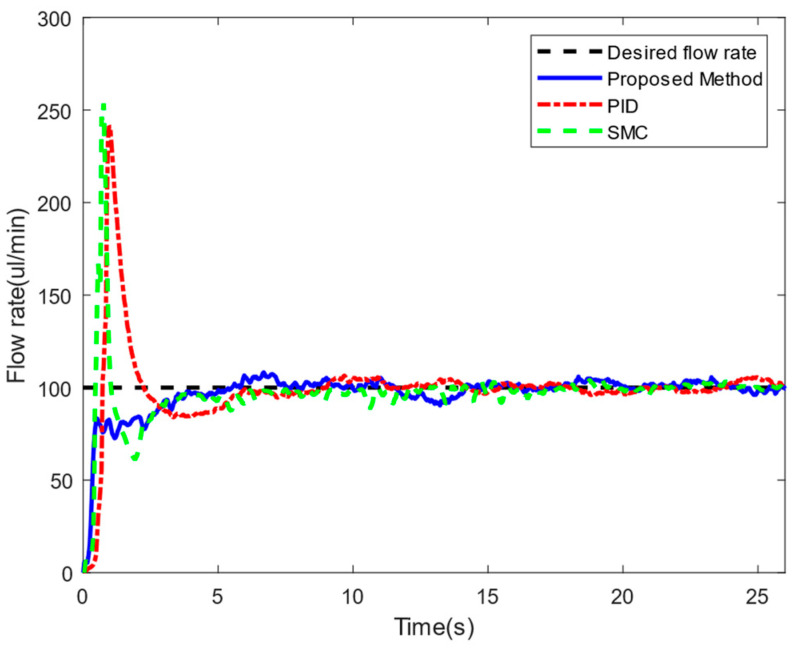
Experimental results (desired value: 100 μL/min).

Firstly, we set the desired flow rate of the drug solution to 100 μL/min and controlled the injection system for flow-rate tracking. As shown in Figure 9, the proposed PID-SMC method was compared with the PID controller and SMC controller.

Since the PID-SMC method integrates the advantages of the smooth control of PID and the insensitivity of SMC to external perturbations, the proposed method eliminates the overshoot due to the transient action of the drug piston.

In addition, the flow rates of 70 μL/min and 200 μL/min were also tracked and controlled. We selected the tracking results of a steady-state process lasting 30 s to compare the accuracy of the flow rate. Table 1 shows the mean absolute error (MAE) and root mean square error (RMSE) of the different methods. The accuracy of the flow rate controlled by the PID-SMC method in the steady-state process was superior to that of the PID and SMC methods, proving that the designed controller has better convergence characteristics.

### 3.3. Injection Comparative Experiment

In this study, we also compared the proposed liquid-driven injection system with the gas-driven injection system, whose injection principle is the same as that used in the standard vitrectomy system. We utilized active high-pressure gas to drive the syringe and carry out the injection of a medicinal solution through constant pressure. Then we calculated and compared the impact force of the injection process on the human fundus tissue.

In order to obtain the same flow rate (100 μL/min) as the injection experiment in this paper, a strong gas with constant pressure was applied to the end of the drug syringe in the gas-driven injection system, and a flow sensor was connected to the injection pipeline to measure the drug flow. The constant pressure corresponding to the flow rate of 100 μL/min by the gas-driven device was obtained to be 15 kPa through many experiments. We repeated the injection 15 times at a constant pressure of 15 kPa and found that there was always a transiently large flow overshoot for each injection. We recorded the maximum value of the overshoot of each injection and obtained the average overshoot value of the flow rate in all 15 injections, which was 490.6 μL/min.

The maximum impact pressure $P_{2}$ can be calculated by using Equation (18) when the maximum flow rate of the injection system is obtained. The maximum impact pressure on the choroid caused by the maximum flow rate of the gas-driven method was 3492.2 Pa, while the maximum impact pressure was 2700.6 Pa, caused by the constant flow rate of the proposed system. The experimental results indicate that the impact pressure on the fundus was reduced by 22.67% in the proposed injection system compared with the uncontrolled gas-driven injection. The fundus damage caused by the high flow rate can be effectively avoided.

## 4. Conclusions

Microinjection is an important part of fundus surgery. The constant pressure injection process of the standard vitrectomy system is not controllable. The overshooting of the large flow rate will cause damage to the fundus tissue. In this paper, a microinjection system is constructed for fundus injection. The injection system, which is driven by liquid pressure, solves the problem of excessive impact on tissues by fundus injection. Moreover, we carry out theoretical modeling of this system according to the direct physical relationship between the piston velocity and the fluid velocity, and we design a PID-SMC method to output a stable flow rate based on the system model. The asymptotic stability of this controller has been proven by using Lyapunov’s theorem. In order to further verify the superiority of the control method, we first built the system model and controller for simulation in MATLAB/SIMULINK. The simulation results showed that the proposed control method is better in terms of response speed and stability compared to the traditional control method. Then, we constructed an experimental scenario of the fundus pressure environment by placing the injection needle into the water at a certain depth. Through relevant surgical studies, we obtained a range of injection flow rates for fundus injections, from which we selected a certain flow rate for step-tracking experiments in the simulated fundus environment. The results show that the proposed control algorithm has the advantages of no overshooting and high control accuracy, compared with the traditional methods of SMC and PID.

In order to verify the superiority of the system, we conducted experiments in comparison with a gas-driven injection device. The gas-driven injection device was found to have an uncontrollable flow rate with a large overshoot. To quantify the damage to the fundus from overshooting, we modeled the fundus for injection pressure analysis. It was found that the impact pressure on the choroid was reduced by 22.67% compared with the gas-driven device injection, which effectively reduced the damage to the tissue caused by the injection.

In fundus surgery, the procedure of withdrawing the needle when the injection is completed may result in misrelease of the drug due to changes in ocular pressure intensity. In the future, we will conduct a modeling study of fundus pressure and improve the control algorithm to prevent drug misrelease. In addition, the silicone oil-flow fluctuation of the system is large, and we will also consider adding a section of gas above the tee of the silicone oil line as a buffer, which could potentially make the injection system perform better.

## Figures and Tables

**Table 1:** Comparison of steady-state tracking results.

Experiments	Performance Metrics	PID	SMC	PID-SMC
70 μL/min	MAE	2.1657	2.3676	1.5497
	RMSE	2.8043	2.7065	1.9504
100 μL/min	MAE	2.4647	2.5205	2.2707
	RMSE	3.1229	3.4250	2.6955
200 μL/min	MAE	7.1198	11.1117	4.1513
	RMSE	9.2001	12.5003	5.2710

## Data Availability

The raw data supporting the conclusions of this article will be made available by the authors without undue reservation.
